# Androgen Deprivation Therapy for Prostate Cancer Did Not Increase the Risk of Retinal Vascular Occlusion: A Population-Based Cohort Study

**DOI:** 10.3390/ijerph19042268

**Published:** 2022-02-17

**Authors:** Hsin-Le Lin, Chia-Yi Lee, Jing-Yang Huang, Po-Chen Tseng, Shun-Fa Yang

**Affiliations:** 1 Department of Ophthalmology, Taipei City Hospital, Renai Branch, Taipei 106243, Taiwan; yvonne11221110@hotmail.com; 2 Department of Ophthalmology, Nobel Eye Institute, Taipei 106074, Taiwan; ao6u.3msn@hotmail.com; 3 Department of Medical Research, Chung Shan Medical University Hospital, Taichung 402367, Taiwan; wchinyang@gmail.com; 4 Department of Special Education, University of Taipei, Taipei 100234, Taiwan; 5 Department of Optometry, University of Kang-Ning, Taipei 114311, Taiwan; 6 Institute of Medicine, Chung Shan Medical University, Taichung 402367, Taiwan

**Keywords:** androgen deprivation therapy, retinal vascular occlusion, prostate cancer

## Abstract

This study aimed to evaluate the effect of androgen deprivation therapy (ADT) on retinal vascular occlusion (RVO) development in patients with prostate cancer, using data from Taiwan’s National Health Insurance Research Database. A total of 1791, 1791, and 3582 patients were enrolled in the prostate cancer with ADT group, prostate cancer without ADT group, and the control group, respectively. The primary outcome was RVO occurrence, according to diagnostic codes. Cox proportional hazard regression was used to determine the adjusted hazard ratio (aHR) and 95% confidence interval (CI) of ADT and other covariates for RVO incidence. After a follow-up interval of up to 18 years, the patients with prostate cancer who received ADT showed significantly lower RVO incidence than the control group (aHR: 0.191, 95% CI: 0.059–0.621, *p* = 0.0059), after adjusting for multiple confounders. Hypertension was related to higher RVO incidence (aHR: 2.130, 95% CI: 1.127–4.027, *p* = 0.0199). Our overall results showed that using ADT for prostate cancer did not lead to a greater risk of RVO development. In fact, the patients with prostate cancer who received ADT had lower RVO incidence than those who did not receive ADT.

## 1. Introduction

Prostate cancer is the second most common cancer among men, and represents the fifth leading cause of cancer death worldwide [1]. Androgen deprivation therapy (ADT) plays a critical role in the management of aggressive and advanced prostate cancer. There are different types of ADT, including surgical castration (bilateral orchiectomy), gonadotropin-releasing hormone (GnRH) agonists or antagonists, antiandrogens, and estrogens. Several randomized trials have indicated improved disease control and overall survival benefit with the addition of ADT to external beam radiotherapy [2,3].

Considering the overall survival improvement after ADT, in patients with advanced prostate cancer, there have been increasing concerns regarding treatment-related side effects that may significantly affect a patient’s quality of life, as well as cause mortality/morbidity. Although reducing the amount of androgens may slow the tumor growth, with rapid and dramatic clinical benefits, it may also impact other organs, causing numerous side effects, including hot flashes, sexual dysfunction, reduced musculoskeletal health, decreased insulin sensitivity, obesity, dyslipidemia, as well as potential harm to cardiovascular and neurologic systems [4]. Macrovascular thromboembolic events, including venous thromboembolism [5], myocardial injury [6], and stroke [7], are common during advanced prostate cancer management, especially in patients receiving systemic therapy. However, microvascular events, such as retinopathy, nephropathy, and neuropathy, have not been well investigated among patients receiving ADT.

The literature reports conflicting data regarding the role of ADT in systemic macrovascular health. For instance, Tsai et al. [8] showed that ADT is associated with an increased risk of death from cardiovascular diseases in patients with localized prostate cancer. However, a meta-analysis revealed that ADT use among patients with an unfavorable risk of prostate cancer was not associated with an increased risk of cardiovascular death, but was associated with a lower risk of prostate cancer-specific mortality and all-cause mortality [9]. Retinal vascular occlusion (RVO) is the obstruction of microvascular circulation in the retina. The abnormality of retinal microcirculation may lead to retinal ischemia, retinal hemorrhages, leakage of fluid from the blocked blood vessels, and, in the end, irreversible loss of vision if left untreated [10]. It is known that RVO and systemic cardiovascular diseases share common risk factors, such as age and other conventional risk factors for atherosclerosis, including hypertension, hyperlipidemia, and DM [11,12]. In this respect, the ocular circulation of prostate cancer patients treated with ADT may be a significant issue in the potential association between ADT and vascular health. To our knowledge, no study to date has reported about the adverse effects of ADT on the vasculature of the eye.

Therefore, the aim of our investigation was to explore the safety of ADT, as reflected by outcomes of retinal vascular occlusion (RVO), in a nationwide cohort study involving patients with prostate cancer, using Taiwan’s National Health Insurance Research Database (NHIRD).

## 2. Materials and Methods

### 2.1. Data Source

This retrospective cohort study was conducted according to the Declaration of Helsinki of 1964 and its subsequent amendment, and approved by both the Institutional Review Board of Chung Shan Medical University Hospital (project identification code: CS1-20108) and the National Health Insurance Administration. Furthermore, the need for informed consent from participants was waived by the two institutions mentioned above. The NHIRD in Taiwan contains the data of health insurance claims for almost the entire Taiwanese population, which includes approximately 23 million individuals. The recruitment interval of the NHIRD was from 1 January 2000 to 31 December 2018, and the available data from the NHIRD include the International Classification of Diseases, Ninth Revision (ICD-9) diagnostic code, the International Classification of Diseases, Tenth Revision (ICD-10) diagnostic codes, demographic data, code of examination, code of procedure, and the ATC codes for prescribed medications. In the present study, we used the Longitudinal Health Insurance Database (LHID), 2005 version, which is a sub-database of the NHIRD. From the LHID 2005, approximately two million individuals were randomly selected from the NHIRD in the year 2005, and the data of these patients were used for all analyses in the present study. Of note, although we drafted the study population in the year 2005, the medical records of the participants we selected can be tracked from 2000 to 2018 because the LHID 2005 includes the medical data from 2000 to 2018, just as the NHIRD does.

### 2.2. Patient Selection

**Figure 1:**
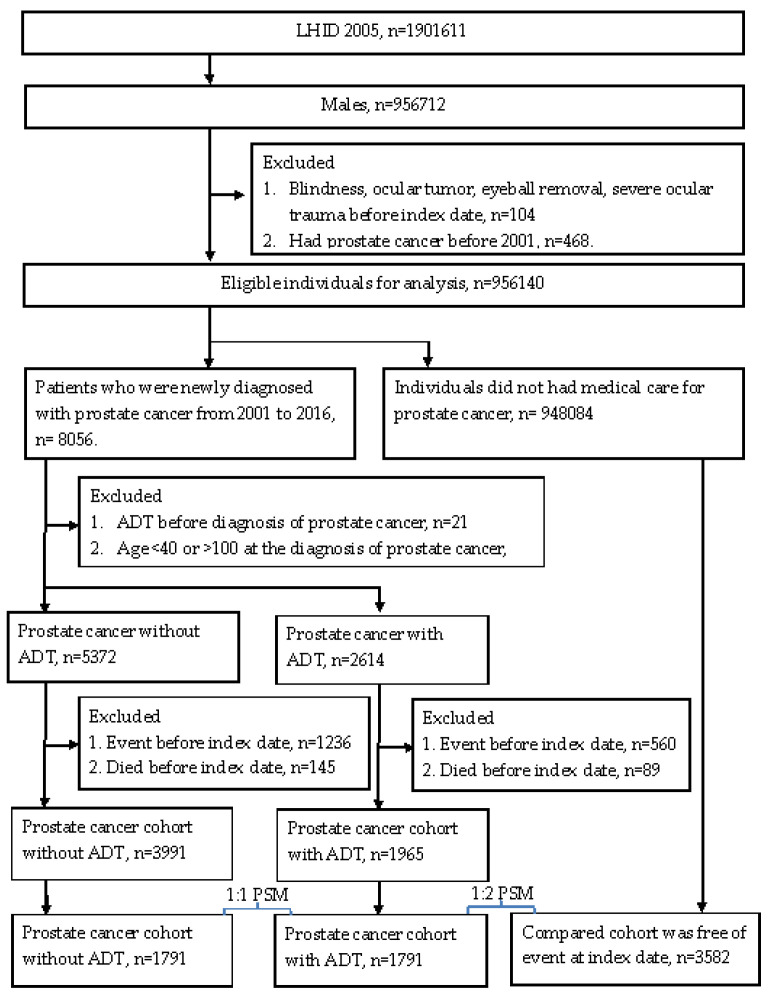
Study flowchart.

Individuals in the prostate cancer with ADT group were enrolled if the data indicated (1) an ICD-9 or ICD-10 diagnostic code of prostate cancer; (2) the arrangement of one of the following treatments: LHRH agonists, antiandrogens, aromatase inhibitors, estrogens, or bilateral orchiectomy, according to the associated procedure codes or ATC codes; (3) male sex; (4) age from 40 to 100 years. For more details, the development of prostate cancer was defined as the receipt of a prostate cancer-related diagnostic code, and the prostate biopsy and alpha-fetoprotein exam needed to be arranged before the appearance of related diagnostic codes. The performance of prostate biopsy as well as alpha-fetoprotein exam was based on the procedure codes. In addition, we excluded males younger than 40 years old because men of this age are rarely diagnosed with prostate cancer in the clinical situation in Taiwan. The index date was defined as 6 months after the initiation of ADT in patients with prostate cancer. To exclude some conditions that may impact the results significantly, the following exclusion criteria were used: (1) blindness before the index date, according to the related ICD-9/ICD-10 diagnostic codes; (2) diagnosis of ocular tumor before the index date, according to the related ICD-9/ICD-10 diagnostic codes; (3) receipt of eyeball removal surgery before the index date, according to the procedure codes; (4) severe ocular trauma, such as eyeball rupture or corneoscleral laceration, before the index date, according to the related ICD-9/ICD-10 diagnostic codes; (5) development of outcome, according to the related ICD-9/ICD-10 diagnostic codes (mentioned in the next section); (6) receipt of ADT before the diagnosis of prostate cancer, according to the related ICD-9/ICD-10 diagnostic codes, procedure codes, or ATC codes; (7) prostate cancer diagnosed before 2001. Then, each patient in the ADT group was matched to one subject with prostate cancer, but without ADT, and two subjects without both ADT and prostate cancer, and the latter served as the control group. The matching process was conducted using the propensity score matching (PSM) process, according to age, socioeconomic status, and education level. If a patient with prostate cancer who was receiving ADT could not be matched to one patient with prostate cancer, but without ADT, and two patients without prostate cancer, that person was removed from the present study. A total of 1791, 1791, and 3582 patients were enrolled in the prostate cancer with ADT group, prostate cancer without ADT group, and the control group, respectively. The process of subject selection is illustrated in Figure 1.

### 2.3. Primary Outcome Measurement

The primary outcome in the present study was the development of RVO, which is considered as (1) the diagnosis of branch retinal venous occlusion, central retinal venous occlusion, branch retinal arterial occlusion, or central retinal arterial occlusion, based on the related ICD-9 or ICD-10 diagnostic codes; (2) the arrangement of fundus examination before the diagnosis of RVO; (3) RVO diagnosed by an ophthalmologist. To examine the possible relationship between ADT and RVO, only RVO that developed after the index date was considered as achievement of the primary outcome in this study.

### 2.4. Demographic and Comorbidity Variables

To render the general status more homogeneous and reduce possible confounders, the effects of the following covariates were included in the multivariable analysis: age, urbanization, insurance type, education level, hypertension, diabetes mellitus (DM), coronary arterial disease (CAD), acute myocardial infarction (AMI), hyperlipidemia, cerebrovascular disease, dementia, and certain retinopathy-related medications, including aminoquinolines, phenothiazines, tamoxifen, desferrioxamine, and nitrofurantoin. The presence of comorbidities was identified according to the related ICD-9/ICD-10 diagnostic codes, and the use of medications was identified based on the ATC codes. Furthermore, CAD indicated those with chronic ischemic heart disease. Each patient was followed up longitudinally from the index date to the date of RVO diagnosis, withdrawal from the National Health Insurance program, or the end of the NHIRD/LHID 2005 record, which was 31 December 2018. To be more clear, we gathered patients in 2005 from the LHID 2005 database, and the LHID 2005 database can trace patients to 31 December 2018, as we do in this study.

### 2.5. Statistical Analysis

All statistical analyses in this study were conducted using SAS version 9.4 (SAS Institute Inc., Cary, NC, USA). After PSM, we used descriptive analysis to demonstrate the baseline characteristics of the three groups. Next, Poisson regression was applied to determine the incidence rate and corresponding 95% confidence intervals (CIs) of RVO among the three groups. In the next step, Cox proportional hazard regression was used to reveal the crude and adjusted hazard ratio (aHR) of RVO among the three groups, which considered the potential effect of demographic data, systemic diseases, and the use of specific medications in the multivariable analysis. Cox proportional hazard regression was also applied to calculate the effect of each covariate on the development of RVO, which presented as aHR again. In particular, the effects of retinopathy-related medications were not estimated, due to the extremely few numbers of usages. Kaplan–Meier curves were plotted to reveal the cumulative probability of RVO among the prostate cancer with ADT group, prostate cancer without ADT group, and control group, and the log-rank test was conducted to evaluate whether a significant difference existed among the three survival curves. The level of statistical significance was set at *p* < 0.05 in this study.

## 3. Results

Table 1 shows the baseline characteristics of the three groups. Age, urbanization, insurance type, and education level were similar among the three groups, due to the PSM procedure. Similarly, the distribution of comorbidities and the use of retinopathy-related medications showed no significant differences among the three groups (Table 1). Regarding the type of ADT used in the prostate cancer with ADT group, management was most commonly performed with antiandrogens, which were prescribed to >67% of individuals in the prostate cancer with ADT group, whereas estrogen was used with the least frequency (Table 1).

**Figure 2:**
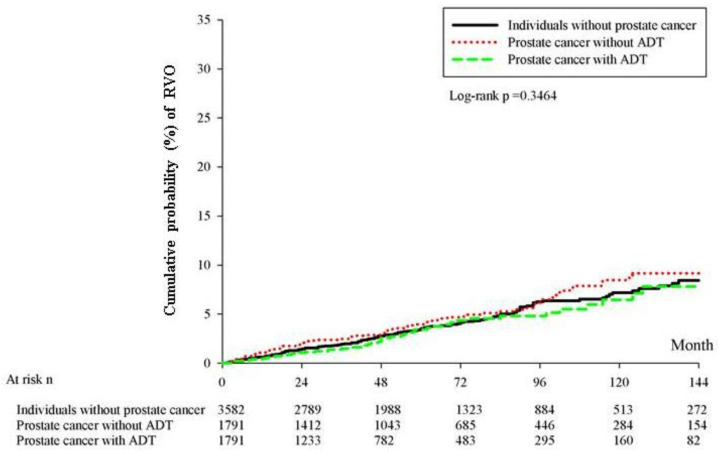
Cumulative probability of crude incidence rates.

After a follow-up interval of up to 18 years, there were 3, 10, and 38 cases of new RVO events in the prostate with ADT, prostate without ADT, and control groups, respectively. The patients with prostate cancer who received ADT showed significantly lower incidence of RVO than the patients in the control group (aHR: 0.191, 95% CI: 0.059–0.621, *p* = 0.0059), after adjusting for multiple potential confounders for RVO (Table 2). However, the cumulative probability of the crude incidence rates showed no significant difference among the three groups (log-rank *p* = 0.3464) (Figure 2). In the analysis of each covariate, the presence of hypertension was associated with a higher rate of RVO development (aHR: 2.130, 95% CI: 1.127–4.027, *p* = 0.0199). Nonetheless, no other covariates exhibited a prominent effect on the development of RVO, according to the multivariable analysis (all *p* > 0.05) (Table 3).

## 4. Discussion

In this large population-based study, our results demonstrated that there were no significant associations among patients between the administration of ADT and the development of RVO, as assessed using the Kaplan–Meier analysis. However, the multivariate Cox regression analysis revealed that the administration of ADT was associated with a decreased hazard of RVO, after adjusting for multiple potential confounders.

The precise mechanism of action of androgen on retinal tissue and the possible thrombotic risks probably involve multiple factors. There is a potential role for androgen in retinal disease. The androgen receptor protein has been identified in endothelial cells [13,14] and retinal tissue [15,16,17,18] in several experimental studies. Moreover, exogenous androgen has been found to induce a proinflammatory effect in vascular endothelial cells via the expression of adhesion molecules [13,14,17]. The enhancement of monocyte adhesion to endothelial cells promotes vascular inflammation and further results in vascular pathologies, including atherosclerosis. One retrospective, matched cohort study, conducted using data from a large national US insurance database, implicated that patients using testosterone supplementation are at greater risk of developing retinal artery occlusion (HR: 1.43, 95% CI: 1.12–1.81, *p* = 0.004) [15]. The effect of androgen supplementation on retinal vessels still remains uncertain.

A wide diversity of opinions exists on ADT and the risk of thromboembolic events. Various studies have linked ADT with an increased risk of cardiovascular and cerebrovascular events. For instance, Teoh et al. [6,7] investigated 452 Chinese men with prostate cancer, and found that ADT administration was associated with increased risks of developing AMI (HR: 6.78, 95% CI: 1.31–35.05, *p* = 0.022) and ischemic stroke (HR: 3.32, 95% CI: 1.14–9.67, *p* = 0.028), after multivariate Cox regression analysis. Another database study, within the United Kingdom’s General Practice Research Database population, analyzed 22,310 patients with prostate cancer and provided evidence that different types of ADT, including GnRH agonists, oral antiandrogens, combined androgen blockade, bilateral orchiectomy, and others, may increase the risk of stroke and transient ischemic attacks [19]. In contrast, several studies have suggested that there is no significant association between ADT administration and thromboembolic events. Chung et al. [20] suggested that there was no significant increase in the risk of ischemic stroke among patients with prostate cancer, who received ADT. The reports of ADT-associated thromboembolic diseases have primarily concentrated on macrovascular events, and there are limited data regarding microvascular events. In short, ADT may damage the vascular structure and lead to thromboembolic events, and ocular thromboembolic events are the etiology for RVO. Accordingly, the relationship between ADT and subsequent RVO should be evaluated.

Our study showed that the use of ADT did not increase the risk of RVO, and was associated with a decreased hazard of RVO, after the multivariate Cox regression analysis. This result is similar to the concept of Kaur et al. [21] that ADT reduces thrombophilic activation in men with advanced prostate cancer and protects against thrombotic risk. Moreover, as hypercoagulability is one of the important risk factors for RVO, it is possible that ADT reduces tumor burden and improves cancer-induced hypercoagulability over time, and, consequently, reduces RVO risk. Similarly, Liao et al. [22] used the NHIRD and found that ADT was not associated with the risk of ischemic stroke in patients with prostate cancer, after adjusting for potential confounders. They also suggested further study to evaluate whether a different thromboembolic mechanism exists at different vasculature sites in patients with prostate cancer, who are receiving ADT. In fact, when reviewing the studies published to date, RVO was not observed, or addressed, to be associated with ADT use. Based on our results, we believe that ADT would not elevate the risk of RVO in patients with prostate cancer and RVO risk factors. Our study had several strengths. The large sample of insured individuals throughout Taiwan allows for a more meaningful study. All the medical management information was obtained from medical records, rather than from patient self-reports, which, thus, eliminated recall bias. Finally, the matched cohort study design allows for a reduction in selection bias.

We also found that hypertension significantly increased the risk of developing RVO. This finding is similar to that of previous studies, which showed that uncontrolled hypertension is a known risk factor for the development of RVO [11]. In addition to hypertension, some studies suggested that other conventional risk factors for atherosclerosis, including hyperlipidemia and DM, play a role in the pathogenesis of RVO [11,12]. In our study, we found no association between RVO and comorbidities, such as hyperlipidemia, DM, and cerebrovascular and cardiovascular diseases. A possible explanation for this is that we used multivariable analysis in this study, and, thus, the effects of these covariates were diminished by the effect of hypertension, whereas the previous study did not apply such an analysis. Further observational studies may be required to better determine the role of these comorbidities in patients with RVO. In regards to the procedures prior to ADT in the prostate cancer with ADT group, the patients who received combined surgical and radical management accounted for most of the cases. Concerning the influence of these procedures on the outcome of ADT, combined therapy may, theoretically, lead to a better outcome. However, because the tumor staging and image cannot be obtained from the NHIRD/LHID, we cannot evaluate the related results in the current study. Moreover, there are few studies that discuss the relationship between these procedures and subsequent/co-existing systemic morbidities, such as vascular or metabolic disorders. Further research may be needed to evaluate the potential effect of these procedures on the therapeutic outcome and co-morbidities.

Our study had several limitations. The claims data from the NHIRD lack details regarding confounding factors, such as smoking status, body mass index, and dietary habits, which may have relevance to the etiology of RVO. Details about other clinical factors, such as the Gleason score, tumor grading, retinal images, and stereotactic fundus pictures, were not recorded in the NHIRD. Therefore, we could not evaluate the severity of RVO or the association between ADT and RVO in different cancer stages. Moreover, we could not evaluate the association between each type of ADT and RVO because some patients received more than one type of ADT. Finally, there may have been some underdetected patients, with asymptomatic RVO, especially those with occlusion of smaller branches.

## 5. Conclusions

The administration of ADT for prostate cancer does not lead to a greater risk of RVO development. The patients with prostate cancer who received ADT had lower RVO incidence than those who did not receive ADT. Therefore, we suggest that physicians should evaluate the possible causes of RVO, other than ADT, in patients with advanced prostate cancer. Furthermore, patients with RVO risk factors are probably not warranted to discontinue ADT. In short, the use of ADT is safe, concerning the risk of RVO, even in patients with risk factors of RVO. Further research is needed to understand the mechanisms underlying the observed associations.

## Figures and Tables

**Table 1:** Baseline characteristics of study population.

Characteristics	Control	Prostate Cancer without ADT	Prostate Cancer with ADT	*p* Value
Age at index date (years)				0.9607
<50	19 (0.53%)	9 (0.50%)	10 (0.56%)	
50–59	220 (6.14%)	101 (5.64%)	107 (5.97%)	
60–69	922 (25.74%)	453 (25.29%)	463 (25.85%)	
70–79	1498 (41.82%)	778 (43.44%)	737 (41.15%)	
≧80	923 (25.77%)	450 (25.13%)	474 (26.47%)	
Urbanization				0.8220
Urban	2017 (56.31%)	995 (55.56%)	982 (54.83%)	
Sub-urban	1160 (32.38%)	580 (32.38%)	596 (33.28%)	
Rural	405 (11.31%)	216 (12.06%)	213 (11.89%)	
Occupation				0.7806
Government employees	279 (7.79%)	138 (7.71%)	139 (7.76%)	
Labor	1336 (37.30%)	661 (36.91%)	657 (36.68%)	
Farmer and fisherman	1047 (29.23%)	553 (30.88%)	529 (29.54%)	
Low income	13 (0.36%)	13 (0.73%)	12 (0.67%)	
Unemployed	855 (23.87%)	401 (22.39%)	428 (23.90%)	
Others	52 (1.45%)	25 (1.40%)	26 (1.45%)	
Education years				0.8728
<6	1987 (55.47%)	989 (55.22%)	971 (54.22%)	
6–9	454 (12.67%)	235 (13.12%)	253 (14.13%)	
9–12	830 (23.17%)	408 (22.78%)	408 (22.78%)	
≥12	311 (8.68%)	159 (8.88%)	159 (8.88%)	
Comorbidities				
Hypertension	1907 (53.24%)	951 (53.10%)	961 (53.66%)	0.9389
DM	637 (17.78%)	336 (18.76%)	360 (20.10%)	0.1182
CAD	567 (15.83%)	287 (16.02%)	316 (17.64%)	0.2185
AMI	17 (0.47%)	10 (0.56%)	13 (0.73%)	0.5072
Hyperlipidemia	616 (17.20%)	291 (16.25%)	326 (18.20%)	0.3010
Cerebrovascular disease	430 (12.00%)	227 (12.67%)	238 (13.29%)	0.3920
Dementia	91 (2.54%)	47 (2.62%)	56 (3.13%)	0.4446
Retinopathy-related medications				
Aminoquinolines	7 (0.20%)	4 (0.22%)	4 (0.22%)	0.9671
Phenothiazines	13 (0.36%)	18 (1.01%)	9 (0.50%)	0.0111
Nitrofurantoin	0 (0.00%)	20 (1.12%)	0 (0.00%)	-
ADT type				-
LHRH agonists			1108 (61.86%)	
Antiandrogens			1212 (67.67%)	
Estrogens			140 (7.82%)	
Bilateral orchiectomy			202 (11.28%)	
Procedure prior to ADT				
Surgery			589 (32.89%)	
Radiotherapy			547 (30.54%)	
Combined surgery and radiotherapy			649 (36.24%)	
None			6 (0.33%)	

DM: diabetes mellitus; CAD: coronary arterial disease; AMI: acute myocardial infarction; ADT: androgen deprivation therapy.

**Table 2:** Incidence risk of study events among the three groups.

Event	Control	Prostate Cancer without ADT	Prostate Cancer with ADT
Follow-up (person month)	236,871	122,287	96,035
New case	38	10	3
Incidence rate # (95% CI)	1.60 (1.17–2.20)	0.82 (0.44–1.52)	0.31 (0.10–0.97)
Crude relative risk (95% CI)	Reference	0.509 (0.254–1.022)	0.196 (0.061–0.636) *
Adjusted HR (95% CI)	Reference	0.525 (0.261–1.056)	0.191 (0.059–0.621) *

# Incidence rate, per 10,000 person-month; * denotes significant difference compared with the control group; CI: confidence interval.

**Table 3:** Effect of each covariate on retinal vascular occlusion.

Covariate	aHR	95% CI	*p* Value
Group			
Control	Reference		
Prostate cancer without ADT	0.525	0.261–1.056	0.0707 *
Prostate cancer with ADT	0.191	0.059–0.621	0.0059 *
Age (years)			
<50	-		
50–59			
60–69	3.566	0.466–27.281	0.2207
70–79	3.099	0.405–23.739	0.2762
≧80	4.107	0.511–32.997	0.1839
Urbanization			
Urban			
Sub-urban	0.981	0.495–1.945	0.9572
Rural	1.184	0.412–3.399	0.7535
Occupation			
Government employees	1.338	0.480–3.728	0.5772
Labor			
Farmer and fisherman	0.757	0.312–1.838	0.5391
Low income	5.238	0.581–47.240	0.1400
Unemployed	0.988	0.456–2.141	0.9749
Others	1.826	0.226–14.764	0.5724
Education years			
<6	1.436	0.546–3.773	0.4632
6–9			
9–12	1.642	0.586–4.598	0.3454
≥12	0.855	0.200–3.652	0.8326
Comorbidities			
Hypertension	2.130	1.127–4.027	0.0199 *
DM	1.600	0.817–3.135	0.1707
CAD	0.770	0.348–1.704	0.5195
AMI	5.279	0.677–41.141	0.1122
Hyperlipidemia	0.390	0.149–1.022	0.0555
Cerebrovascular disease	1.187	0.543–2.597	0.6671
Dementia	1.070	0.143–8.003	0.9475

* denotes significant effect on the development of RVO after adjusting for the influence of all covariates; CI: confidence interval; ADT: androgen deprivation therapy; DM: diabetes mellitus; CAD: coronary arterial disease; AMI: acute myocardial infarction.

## Data Availability

The data presented in this study are available, on request, from the corresponding author.
